# Supplementary material for: Downstream network transformations dissociate neural activity from causal functional contributions
Source: Sci Rep. 2024 Jan 24;14:2103. doi: 10.1038/s41598-024-52423-7 (PMC10808222; doi:10.1038/s41598-024-52423-7)
Supplement: Supplementary file 1 — Supplementary Figures. [file 41598_2024_52423_MOESM1_ESM.pdf]

Supplementary Figures:

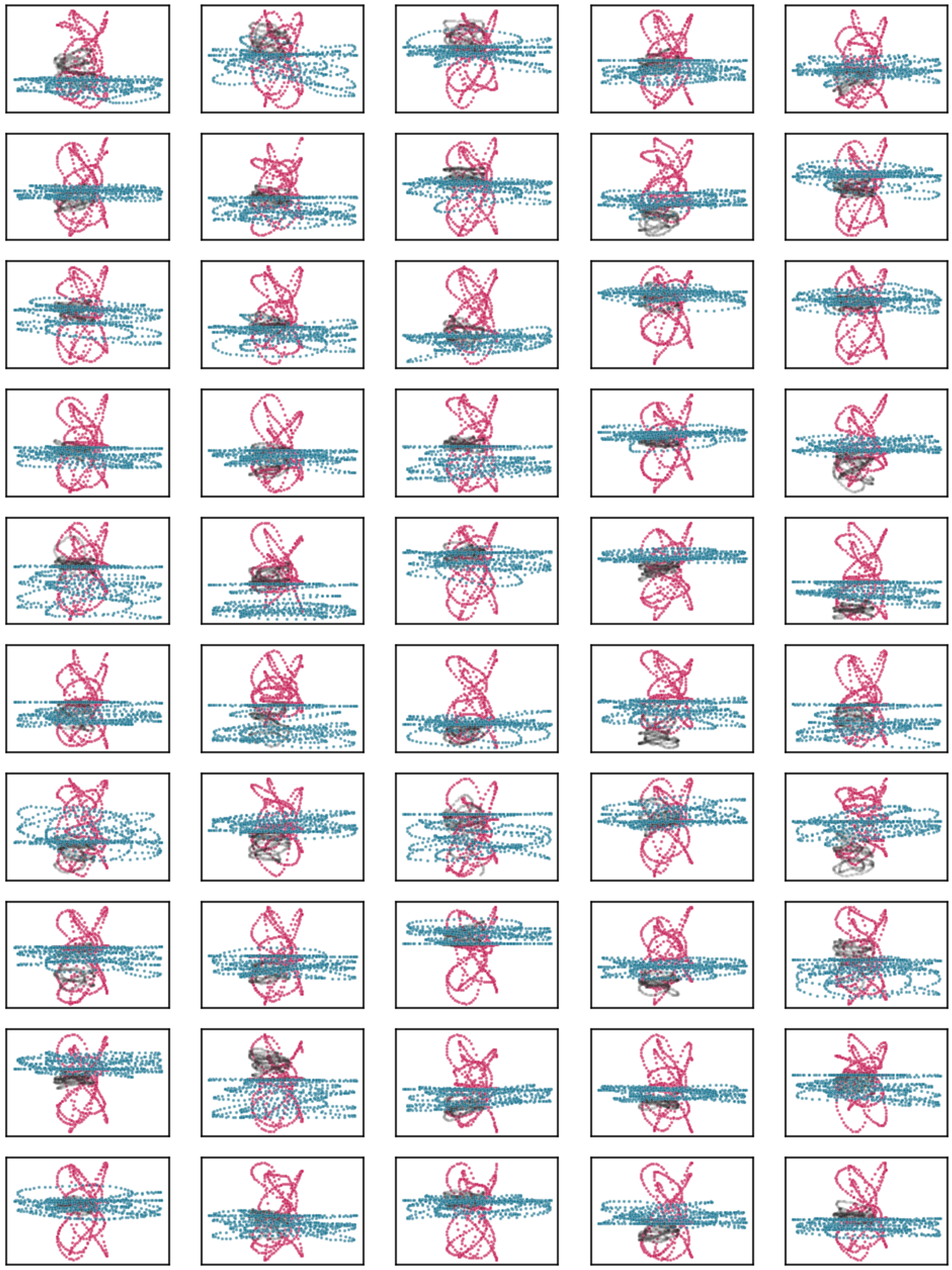

Supplementary Figure 1: Testing with 50 different MSA random initialization. Colors and axes follow Fig. 5 with median weight plotted in black for clarity.

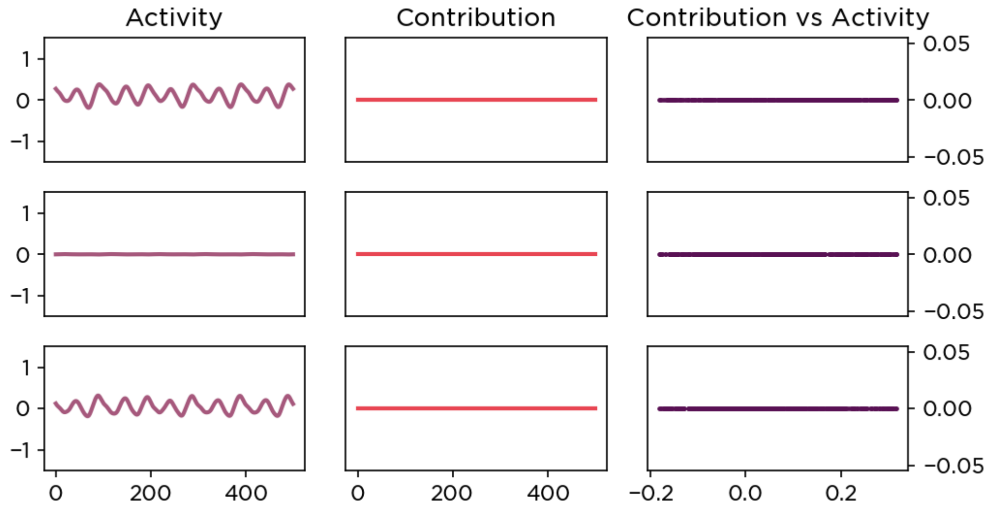

Supplementary Figure 2: Playing games without perturbation. Activity profiles and causal contributions of the same three example nodes as in Fig.4 are plotted for comparison. Note that the contributions are all zero.

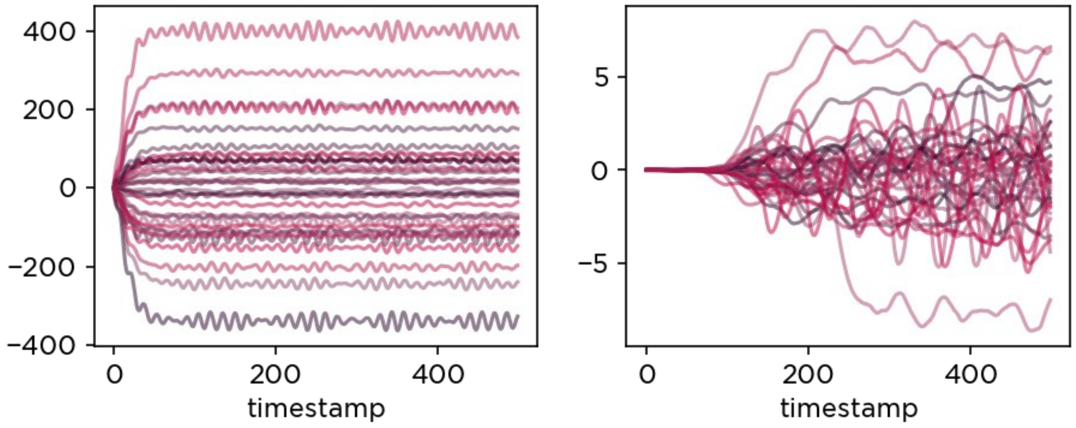

Supplementary Figure 3: Playing without retraining the readout layer. Causal contributions of all 36 nodes in the hidden layer. On the left, the fine-tuning step was omitted, and thus the network became dysfunctional given any small perturbations (note the y-axis). In right, the readout weights were allowed to adjust to the disrupted dynamics, and thus, the network could still generate a reasonable output signal.

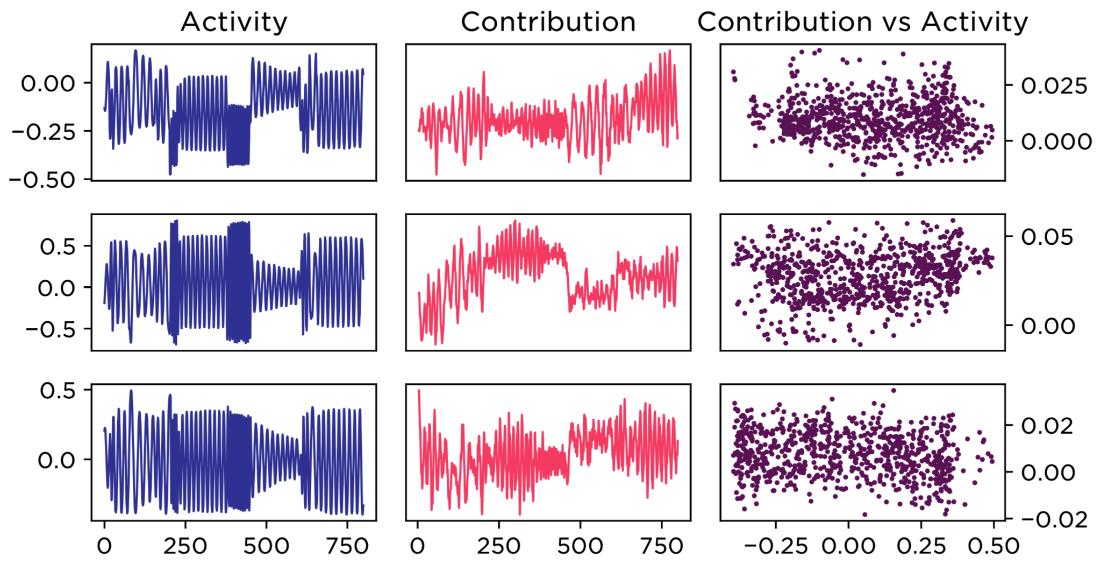

Supplementary Figure 4: Contributions and activity profiles of a separate network solving a frequency generator task. Activity profiles and causal contributions of three example nodes in a separate network are plotted for comparison. As with the main network, the relationship between a node's contribution and its activity profile is nontrivial.
